# To: Epistaxis as a complication of high-flow nasal cannula therapy in adults

**DOI:** 10.5935/0103-507X.20220047-en

**Published:** 2022

**Authors:** Abhijit Nair, Antonio Esquinas

**Affiliations:** 1 Department of Anesthesiology, Ibra Hospital - Ibra, Oman.; 2 Department of Intensive Care, Hospital Morales Meseguer - Murcia, Spain.

## TO THE EDITOR

We have read with great interest this original article by Veiga et al. titled “Epistaxis as a complication of high-flow nasal cannula therapy in adults”.^(1)^ Although epistaxis is an infrequent complication, it is very interesting as epistaxis has important clinical repercussions. The authors consider that the high flow rate in use (65L/minute) and smaller prong configuration that increases the velocity of the gas represent a jetting effect. Although the authors did not find any difference in risk factors for epistaxis, we propose several factors that should be taken into account.

First, information about the associated mechanism and mucosal tissue of the nasal airways is essential. From a physiological point of view, we do not have information about the impact of mouth breathing prevalence. Mouth breathing is a critical factor for humidity and temperature control at the nasal-mouth level.^(2,3)^ We consider that the fundamental mechanism is a loss or ineffectiveness of humidity, which is related to nasal dryness together with the added effects of oxygenation.^(4)^

Second, information about epistaxis evaluation and treatment (otorhinolaryngologic assessment) is essential. Epistaxis is a sign and requires more precise objective evaluation and treatment.

Some grading or type of score (Epistaxis Severity Score) that is validated for hereditary hemorrhagic telangiectasia can be useful.^(5)^

Performing an exploration of the mucosa or nasal cavity by utilizing anterior rhinoscopy to identify whether patients with epistaxis have unilateral or bilateral injury can be helpful.

It is important to know what treatment was offered for epistaxis, such as cauterization (either chemical with trichloroacetic acid or electrical with bipolar forceps) along with nasal packing.

It is also important to know whether, in addition to epistaxis as a sign, there are other associated symptoms, such as nasal obstruction, pain, mucosal injury, crusting, rhinorrhea, nasal twang in speech, hyposmia, and breathing difficulties.

Third, information on the accuracy of the nasal high flow system is also important. The authors used the Vapotherm^®^, Inc., Exeter, nasal high flow system, which has small-bore nasal cannulas (sizes 2.7mm and 4.8mm). Although the temperature was adjusted between 35°C and 37°C, it is important to know that some bench models of nasal high flow system devices can lose temperature-humidity stability.^(5)^ In addition, the external temperature of the intensive care unit can have an impact.

These factors could aid in understanding epistaxis and in selecting a rational approach for its treatment in patients with nasal mucosa frailty.
